# A comparison of the effects of electroacupuncture vs transcutaneous electrical nerve stimulation for pain control in knee osteoarthritis: A protocol for network meta-analysis of randomized controlled trials

**DOI:** 10.1097/MD.0000000000016265

**Published:** 2019-07-12

**Authors:** Xiaowei Shi, Wenjing Yu, Tong Wang, Qi Shu, Chunjiu Wang, Xue Yang, Changxin Liu, Changqing Guo

**Affiliations:** a School of Acupuncture-Moxibustion and Tuina; b Department of pediatrics, Beijing University of Chinese Medicine Third Affiliated Hospital; c Department of Tuina and Pain, Beijing University of Chinese Medicine Dongzhimen Hospital, Beijing University of Chinese Medicine, Beijing, China.

**Keywords:** electroacupuncture, knee osteoarthritis, network meta-analysis, pain, transcutaneous electrical nerve stimulation

## Abstract

**Background:**

Knee osteoarthritis (KOA), the most common type of osteoarthritis, is a chronic degenerative joint disease accompanied by pain and functional limitation for the elderly. The 2 nonpharmacologic approaches, electroacupuncture (EA) and transcutaneous electrical nerve stimulation (TENS), are considered beneficial in relieving KOA pain, however, the current conclusions are controversial. Furthermore, no direct or indirect meta-analyses between EA and TENS have been reported for the pain relief of KOA patients.

**Methods:**

PubMed, EMBASE, Cochrane library, Web of Science, CNKI, VIP, Wan Fang will be systematically searched their inception to May 2018. Randomized controlled trials that compared the effect of EA and TENS on pain control in knee osteoarthritis will be included. The primary outcome was the knee pain levels, and secondary outcome was the comprehensive indicators. Risk of bias assessment of the included studies will be performed according to the Cochrane risk of bias tool. The pairwise and network meta-analysis will be performed by STATA 14.0 software.

**Results:**

This study is ongoing and will be submitted to a peer-reviewed journal for publication.

**Conclusion:**

This study will provide comprehensive evidence on the effects of EA and TENS for pain control in knee osteoarthritis.

**PROSPERO registration number:**

CRD42018091826.

## 1 Introduction

Osteoarthritis (OA) is the most common joint disease which affects approximately 250 million people worldwide.^[1]^ In developed countries, economic resources allocated to the management of OA accounts for 1.0% to 2.5% of gross domestic product.^[2]^ Kotlarz estimated that the costs due to absenteeism associated with osteoarthritis was $10.3 billion each year from national Medical Expenditure Panel Survey (MEPS) data.^[3]^ Knee osteoarthritis (KOA) represented about 83% of the total OA burden.^[4]^ More than 9,000,000 people have been diagnosed with KOA through clinical and imaging methods in USA.^[5]^ KOA is a chronic disease based on degenerative joint disorder leading to pain and functional limitation, and probably the leading cause of disability in adults.^[6]^ Pain caused by KOA is an important outcome in the progression of the disease because it limits the mobility of patients, cause psychosocial problems such as low self-efficacy and depression,^[7]^ and decreases the quality of life. Thus the current therapeutic approaches for KOA are primarily aimed at alleviating joint pain and slowing its progression.^[8]^ Yet, the prevalence of KOA is constantly increasing,^[9]^ and the only effective pain management approach for the end-stage KOA is knee replacement surgery.^[10]^ With the improved understanding of the pathogenesis and assays of disease activity, researchers are now focusing on the prevention and early treatment of osteoarthritis.^[11]^

Recent guidelines on the non-surgical treatment of knee OA involve patient education and lifestyle self-management, nonpharmacologic and pharmacologic management. Pharmacologic treatments are widely adopted in clinical practice, including acetaminophen, topical treatment, nonsteroidal anti-inflammatory drugs (NSAIDs), COX-2 inhibitors, opioids, and intra-articular cartilage-protective agents (e.g., glucocorticoids).^[12–14]^ However, anti-inflammatory drugs associated with serious adverse reactions in some patients limits the clinical application. Comparison with the pharmacologic treatment, the guidelines for KOA are more conservative nonpharmacologic approaches,^[13,14]^ which are central to managing chronic knee pain.^[15]^ Transcutaneous electrical nerve stimulation (TENS), one of nonpharmacologic treatments, is a neuromodulation therapy proposed by Melzack in 1965.^[16]^ After which, many types of TNES were invented and widely used in many pain managements such as knee pain,^[8]^ due to its low price and simplicity. One research reported that the high-frequency TENS (H-TENS) showed better curative effect in pain control of KOA.^[17]^ Acupuncture, another nonpharmacologic treatments, is the most popular alternative for medicine, and is frequently used for patients with joint pain and arthritis in the USA.^[18]^ Electro-acupuncture (EA) combines traditional acupuncture which has been used for decades with electrical stimulation by attaching an electrode to pairs of needles. One study showed that EA produced greater analgesic effects for different types of pain in comparison with manual acupuncture (MA),^[19]^ a Cochrane review also verified the same result of EA in KOA.^[20]^ Furthermore, one researcher argued that EA and MA treatments were not interchangeable and thus need to be separately studied.^[21]^ Compared with traditional acupuncture or manual acupuncture, the application of EA in KOA analgesia needs more attention.

At present, the different guidelines for KOA are controversial about the use of TENS and acupunture/EA, and do not separate traditional acupuncture from EA in the treatment approach. From another point of view, EA and TENS belong to electrical stimulation treatment, but the better intervention for pain control in knee osteoarthritis is still debatable. Although several meta-analyses have been conducted to validate the effectiveness of TENS or EA in knee osteoarthritis,^[17,22–24]^ there is no relevant review and meta-analyses between TENS and EA due to the lack of head-to-head randomized controlled trials (RCTs). Bayesian network meta-analysis is a relatively new evidence generation method which combines all direct or indirect evidences from different treatment comparisons to enable a unified, coherent analysis of all trials.^[25–27]^ Therefore, the aim of this network meta-analysis is to assess the comparative efficacy of EA and TENS for KOA in pain management, especially the role of EA.

## 2 Methods

### 2.1 Study registration

The proposed systematic review will be conducted and reported in accordance with the Preferred Reporting Items for Systematic Reviews and Meta-Analyses Protocols (PRISMA-P) statement guidelines.^[28]^

This systematic review and network meta-analysis protocol has been registered on the PROSPERO 2018 (ID: CRD42018091826).

### 2.2 Study eligibility criteria

#### 2.2.1 Type of studies

Only randomized controlled trials (RCTs) containing EA or TENS against another or against placebo/sham in patients with knee osteoarthritis will be included in this review. Non- randomized studies or patients after knee replacement will be excluded.

#### 2.2.2 Type of participants

Studies that enrolled patients of any age, gender or ethnicity with a clinical diagnosis of knee osteoarthritis will be included.

#### 2.2.3 Type of interventions

We will consider studies evaluating the following treatments: any type of EA or TNES used as the sole treatment for KOA, and compared control comparators such as pharmacological treatment, manual acupuncture or no treatment/ placebo, which act as vital links for the incorporation of indirect evidence in the networks.

#### 2.2.4 Types of outcome measures

##### 2.2.4.1 Primary outcomes

Knee pain levels will be assessed by the visual analogue scale (VAS),^[29]^ the Western Ontario and McMaster Universities Osteoarthritis Index (WOMAC) score pain subscale^[30]^ or the 11-point numeric rating scale (NRS).^[31]^

##### 2.2.4.2 Secondary outcomes

Comprehensive indicators will be assessed by the WOMAC total scores or the 36-item Short-Form Health Survey (SF-36)^[32]^ for quality of life (QOL). In addition, relevant adverse events will be recorded.

### 2.3 Search strategy

We will electronically search the following databases from their inception through May 2018: PubMed, Embase, Cochrane library, Web of science, the China National Knowledge Infrastructure (CNKI), VIP Information (VIP) and Wanfang Data (WAN FANG). A combination of terms of Medical Subject Headings (MeSH) and keywords will be used in the search strategy, including EA or TENS against another or against placebo/sham. The search words in the Chinese databases have the same meaning as those used in the English databases. To ensure that the most recent trials will be included, we will also retrieve unpublished protocols and summary results through a search of the clinical trial registry at https://clinicaltrials.gov/. In addition, we will search the previously published reviews and meta-analysis related to KOA using EA or TENS. There will be no language restrictions in this review.

### 2.4 Identification of studies

The search results from above 7 databases will be imported to ENDNOTE X7 software to data management. Before the literature selection, the study criteria will be conducted between the reviewers to ensure high interrater agreement. After that, 2 reviewers (Wenjing Yu and Tong Wang) will independently evaluate the title and abstract of all studies for possible candidates. Any duplicate studies will be removed. After passing the title and abstract screening, the full-text copies of all eligible studies will be downloaded for re-evaluation. If the reviewer is uncertain about the eligibility of any study, its full text will be obtained to reexamine. An additional reviewer (Qi Shu or Chunjiu Wang) will be consulted in case of disagreement. Excluded studies and the reasons of exclusion will be recorded.

### 2.5 Data collection

After identification of the target RCTs, 2 independent reviewers (Wenjing Yu and Tong Wang) will extract the necessary data from the included RCTs using a customized form created by Microsoft Excel 2010. One reviewer (Xiaowei Shi) will check the accuracy and consistency of all extracted data. The following data will be extracted:

1. the general information of study such as the first author, year of publication, country, groups, sample size, age, sex;
2. the detailed treatment information such as diagnostic criteria, parameters of intervention including the number of treatment sessions and the lasting time for each session;
3. pain scores. Other outcome measurements such as WOMAC total scores or SF-36 score will be extracted if the study is involved.

We will contact the original study authors for missing data whenever possible. Only the available data will be included if it is not possible to acquire the missing data.

### 2.6 Quality of evidence assessment

According to Grading of Recommendations Assessment Development and Evaluation (GRADE), we will assess the quality of evidence as 4 levels: high quality, moderate quality, low quality, and very low quality.^[33]^ In addition, we will use the online guideline development tool (GDT) to conduct this process.

### 2.7 Risk of bias assessment

The Cochrane Risk of Bias tool,^[34]^ which contains 7 specific domains: sequence generation, allocation concealment, blinding of participants and personnel and other aspects of bias, and the risk of bias of all included RCTs will be assessed with methodological quality as low risk, high risk, or unclear risk of bias. If any domain is scored high/low risk of bias, the study will be considered high/low risk of bias. Two reviewers (Qi Shu and Chunjiu Wang) will complete the easement of risk of bias separately. The conflicts or any discrepancies will be resolved by discussion or will be judged by other reviewer (Xue Yang) to achieve the consensus.

### 2.8 Statistical analyses

Network meta-analysis is a statistical method used to synthesize evidence from a network of trials involving the availability of both direct and indirect data for comparisons of interest.^[27,35]^

First, we will conduct classic pair-wise meta-analyses to synthesize studies with the same pair of interventions by using REVIEW MANAGER Software (version 5.0; the Cochrane Collaboration, Oxford, UK). The results will be reported as standard mean differences (SMD) with the corresponding 95% confidence interval (CI). Chi square test and I^2^ test were used to assess heterogeneity across studies.

Second, we will perform the Bayesian network meta-analysis for assessing the therapeutic effect among EA and TENS and other treatments in KOA using STATA software (version 14.0, StataCorp). We will use Markov Chains Monte Carlo method to conduct this network meta-analysis. Node splitting method will be used to evaluate the inconsistency between direct and indirect comparisons. According to the quantitative estimation, we will adjust the inclusion of studies and ultimately obtain an ideal network with consistency. In addition, a sensitivity analysis will be conducted to examine the impact of low methodological quality and small sample size on the overall effect sizes.

## 3 Discussion

The 2 nonpharmacologic approaches, EA and TENS, are widely used to pain management in knee osteoarthritis, although the recommendations among different guidelines are controversial. For example, the National Institute for Health and Care Excellence (NICE) recommends TENS but against the use of acupuncture for KOA.^[36]^ While the American College of Rheumatology (ACR) conditionally recommends TENS and acupuncture. EA is a comprehensive treatment which combined traditional acupuncture and electrical stimulation. One study showed that EA is more effective than manual or traditional acupuncture, and electrical stimulation via skin patch electrodes (e.g., TENS) is as effective as EA.^[19]^ Furthermore, some researcher argued that EA and manual acupuncture (MA) treatments were not interchangeable and thus needed to be separately studied.^[21]^ Thus, this network meta-analysis will provide a detailed summary and analysis of the latest evidence focusing on EA and TENS as well as relevant other treatments for pain management in KOA. Nonetheless, we hope that our findings will assist patients, clinicians and healthcare policy-makers to make a better choice of treatments in KOA, especially the application of EA.

## Author contributions

**Conceptualization:** Xiaowei Shi, Wenjing Yu.

**Data curation:** Xiaowei Shi, Wenjing Yu, Tong Wang, Qi Shu, Chunjiu Wang.

**Funding acquisition:** Xiaowei Shi, Changqing Guo.

**Methodology:** Xiaowei Shi, Changxin Liu.

**Software:** Wenjing Yu, Tong Wang.

**Supervision:** Qi Shu, Chunjiu Wang, Xue Yang.

**Validation:** Changxin Liu, Changqing Guo.

**Visualization:** Wenjing Yu.

**Writing – original draft:** Xiaowei Shi, Wenjing Yu.

**Writing – review & editing:** Tong Wang, Changxin Liu, Changqing Guo.

Xiaowei Shi orcid: 0000-0003-1486-7986.
